# Metabolomic Identification in Cerebrospinal Fluid of the Effects of High Dietary Cholesterol in a Rabbit Model of Alzheimer’s Disease

**Published:** 2012-03-29

**Authors:** Qing Yan Liu, Erin J. Bingham, Susan M. Twine, Jonathan D. Geiger, Othman Ghribi

**Affiliations:** 1 Neurobiology Program, Institute for Biological Sciences, National Research Council of Canada, Ottawa, Ontario, K1A 0R6, Canada; 2 Faculty of Medicine, University of Ottawa, Ottawa, Ontario, Canada; 3 Phenomenome Discoveries Inc. Saskatoon, SK, Canada S7N 4L8; 4 Department of Pharmacology, Physiology and Therapeutics, School of Medicine and Health Sciences, University of North Dakota, Grand Forks, North Dakota

**Keywords:** Metabolomics, Cerebrospinal Fluid, Cholesterol, Alzheimer’s disease, Rabbit Model

## Abstract

**Background:**

Alzheimer’s disease (AD) is the most common neurodegenerative disorder, manifesting clinical symptoms of cognitive impairment and dementia. The vast majority of cases are late onset AD (LOAD), which are genetically heterogeneous and occur sporadically. The neuropathological changes of LOAD can be reproduced by supplementing a rabbit’s diet with 2% cholesterol for 12 weeks.

**Methods:**

In the present study, a non-targeted Fourier transform ion cyclotron resonance mass spectrometry based metabolomics approach and multivariate statistics were used to survey the effect of cholesterol on cerebrospinal fluid metabolites over a 12 week time-course.

**Results:**

Of the 6515 accurate masses detected in the rabbit CSF, 375 showed significant differences in intensity (p < 0.05) between samples collected at different time points. Further analysis of top 95 (p < 0.01) revealed four clusters of metabolites with different expression patterns throughout the course of the cholesterol treatment. The majority of effects were observed in 12 weeks of cholesterol treated samples, while certain masses showed transient changes at 8 weeks but returned back to near the levels of the controls at 12 weeks. The masses that started to change 8 weeks into the treatment may represent early metabolic changes linked to certain defects in the brain related to AD development. Putative metabolite identifications revealed certain phosphorylated glycerolipids and peptide fragments decreased after 8 weeks of cholesterol treatment.

**Conclusion:**

This study showed that there are specific metabolic perturbations which occur in the CSF as a result of high cholesterol loading. Given the changes of short peptide fragments in particular, the effects are likely the consequence of brain degeneration caused by high cholesterol levels. Further investigations of these masses will lead to a greater understanding of the metabolic mechanisms associated with cholesterol-related AD development. Some of these masses may be used as candidates for the development of diagnostic, prognostic or theranostic markers.

## Introduction

Alzheimer’s disease (AD) is a progressive brain-destructive disease that manifests clinical symptoms of cognitive impairment and dementia, and has no reliable method for early detection or cure. Neuropathologically, AD is characterized by the deposition of amyloid β (Aβ) leading to the development of senile plaques and hyperphosphorylated tau protein aggregates within the cortical neurons that form neurofibrillary tangles (NFTs) [1]. Our current understanding of early-onset (familial) AD is derived primarily from studies on genes or gene products identified in a genetically-determined fashion. Three genes have been definitively implicated in the etiology of early-onset AD; mutations of the *amyloid beta precursor protein* (*A*β*PP*) gene and the *presenilin 1* and *2* genes (*PSEN1*, *PSEN2*) cause rare Mendelian forms of the disease [2]. Although these discoveries have been helpful in elucidating the basic molecular pathogenesis of familial AD, they only represent a relatively small fraction of the AD population. The large majority of cases are late onset AD (LOAD), which are genetically heterogeneous and occur sporadically [3]. Only *apolipoprotein E4* (*APOE4*) has been established unequivocally as a susceptibility gene for LOAD [4]. Two recent large-scale Genome-Wide Association Studies [5] in large patient cohorts have identified clusterin, also known as apolipoprotein J, as being independently associated with LOAD. Both ApoE and ApoJ are involved in lipid metabolism/homeostasis [6] as components of HDL [6,7]. Epidemiologically, altered cholesterol metabolism has been linked to increased Aβ production and AD pathogenesis [8–11]. Hypercholesterolemia is an early risk factor for AD, while decreased prevalence of AD is associated with the use of cholesterol-lowering drugs (statins) that inhibit 3-hydroxy-3-methylglutaryl coenzyme A reductase [9–11].

There is evidence that the pathological changes in dementia-causing AD begin decades before the appearance of the first clinical manifestation [12]. Disease-modifying treatments might be most effective when initiated early in the course of AD, before amyloid plaques and neurodegeneration become widespread. Therefore, identifying predictive/diagnostic markers that can detect preclinical changes at the earliest stages is the key for finding effective treatments. Although mild cognitive impairment (MCI) is often used as a transition state between normal aging and dementia, a substantial number of MCI patients are reclassified as normal aging on follow up [13]. The description of MCI has been derived from clinical and neuropathological settings and its definition is continually being revised. Much of our understanding of disease progression of AD has been driven by animal models, which enable controlled studies of physiopathology and biomarker identification as well as allow for the differentiation between causes and consequences. While the usefulness of transgenic (Tg) mice is undisputed in pre-clinical studies, Tg mice fundamentally represent the familial subtype of AD. In contrast, there is a paucity of models for LOAD and it has been suggested that rabbits fed a cholesterol-enriched diet models this disorder [14,15]. The rabbit Aβ peptide sequence is identical to human. When rabbits are fed a diet supplemented with 2% cholesterol alone, or 1% cholesterol plus trace amounts of copper in drinking water, they develop AD pathology. This includes cortical amyloid deposits, and up to twelve other pathological markers also seen in human AD brains [14–18]. Aβ immumoreactive neurons are observed in the hippocampus and adjacent cortex after 4 weeks of the cholesterol enriched diet, and in the frontal cortex after 6 weeks. ApoE immunoreactivity and fully activated microglial cells are observed in rabbits fed the cholesterol diet for 8 weeks [14]. After 10 weeks, soluble Aβ becomes detectable (50–100 pg/mg) by ELISA in the hippocampus, reaching 100–150 pg/mg by 12 weeks, when insoluble Aβ appears (Ghribi unpublished results) along with hyperphosphorylated tau. Significant neuronal loss is observed in the frontal cortex, hippocampus and cerebellum [14–18]. High cholesterol content in neurons is accompanied by an increase in BACE1 activity and a shift of AβPP processing in favour of Aβ production [17]. Despite their potential utility in LOAD research, this model remains more costly to maintain and is less well characterized compared to ‘gold standard’ transgenic rodent models.

In the present study, we have used Fourier transform ion cyclotron resonance mass spectrometry (FTICR-MS)-based metabolomics to obtain an unbiased survey of the metabolomic effects of cholesterol in the etiology of AD-like progression using cerebrospinal fluid (CSF) collected from a time-course during the cholesterol diet treatment. This ‘non-targeted’ approach has the advantage of detecting novel compounds and is therefore ideally suited for biomarker-driven discovery applications. The identification of a metabolomic signature in this model may be subsequently applied to human AD research and may facilitate pathway, network analysis and identification of possible candidate biomarkers for downstream targeted analyses.

## Materials and Methods

### Experimental animals and laboratory procedures

New Zealand white male rabbits (1.5 year old, weighing 3–4 kg) were used in this study. Animals were randomly assigned to two groups as follows: group 1 was fed normal chow (*n* = 3), and group 2 was fed chow supplemented with 2% cholesterol (*n* = 9), (Harlan Teklad Global Diets, Madison, WI). Diets were kept frozen at −10°C to reduce the risk of oxidation. The animals were allowed water filtered through activated carbon filters. Animals were euthanized with 1 ml intravenous injections of euthasol. Cholesterol-treated rabbits were euthanized (three each time point) at 4, 8 and 12 weeks and control rabbits were euthanized (one each time point) at 4, 8 and 12 weeks. Since the average life span of indoor New Zealand white rabbits is 10–12 years, the control rabbits sacrificed along with treated rabbits at different time points were considered similar in age. Cerebrospinal fluid was collected by puncturing a 25 G needle into the cisterna magna. Unfortunately, we had to discard CSF samples from the 4 week control rabbit and one of the 8 week cholesterol-fed rabbit due to visible blood contamination, resulting in a final list of 2 control and 8 treated CSF samples. The samples were centrifuged at 4500 × *g* at 5°C for 5 min to obtain cell free supernatants. The supernatants were divided in 500 µL aliquots, and frozen immediately in liquid nitrogen and stored at −80°C until taken for analysis. At necropsy, animals were perfused with Dulbecco’s phosphate-buffered saline at 37 °C and the brains were promptly removed. All animal procedures were carried out in accordance with the U.S. Public Health Service Policy on the Humane Care and Use of Laboratory Animals and were approved by the Institutional Animal Care and Use Committee at the University of North Dakota.

### Quantification of Aβ levels by ELISA

The control and 12 week cholesterol treated rabbits described above plus 4 addition control and 3 12-week cholesterol treated rabbits from a previous study were used for this experiment [19]. Aβ40 and Aβ42 levels were quantified in the cortex of all animals using an ELISA kit from Biosource (Carlasbad, CA) as per the manufacturer’s protocol and as we have described previously [19]. The values of Aβ levels obtained by ELISA were normalized to the amount of protein in the samples. The values were expressed as means ± standard deviation. The changes in the levels of Aβ were considered significant at p < 0.05. Levels of Aβ40 and Aβ42 were expressed as pg/mg of protein.

### Thioflavin staining

Coronal frozen sections (30 µm), cut at the level of the hippocampus from controls and cholesterol-treated rabbits (12 weeks) and kept in PBS, were incubated in 0.25% potassium permanganate for 20 min, rinsed in 2 × 2 min dH_2_O and then incubated in bleaching solution (1 g potassium metabisulfite, 1 g oxalic acid, in 100 ml dH_2_O) until they appeared white. Section were washed in dH_2_O, allowed to float in 0.25% acetic acid, washed again in dH_2_O, mounted on slides and allowed to dry. Section were again washed in dH_2_O, stained for 5 minutes with a solution of 0.015% Thioflavin-S in 50% ethanol, and briefly rinsed in 2 changes of 50% ethanol followed by 2 brief changes of dH_2_O, and then mounted with glycerin gelly and visualized under a Leica fluorescence microscope.

### Sample extraction

After the addition of tracking standards, CSF samples were prepared for FTICR-MS analysis by sequentially extracting three-time 200 µL of CSF with equal volumes of 1% ammonium hydroxide and ethyl acetate (EtOAc). All extractions were performed on ice. Samples were centrifuged between extractions at 4°C for 10 min at 3500 rpm and the organic layer was removed and transferred to a new tube (extract A). A 1:5 ratio of EtOAc (extract A) to butanol (BuOH) was then evaporated under nitrogen to the original BuOH starting volume (extract B). Aqueous molecules were isolated from extract A using 0.33% formic acid (extract C). All extracts were stored at −80°C until FTICR-MS analysis.

### FTICR-MS analysis

The prepared extracts were analyzed as previously described [20] using electrospray ionization (ESI) (positive and negative) and atmospheric pressure chemical ionization (APCI) (positive and negative) each with organic and aqueous phases of the extraction. Samples were directly injected using ESI and APCI at a flow rate of 600 µL per hour. Collectively, six separate injections were performed on each sample (Table 1). All analyses were performed on a Bruker Daltonics APEX III FTICR-MS equipped with a 7.0 T actively shielded superconducting magnet (Bruker Daltonics, MA, USA). Ion transfer/ detection parameters were optimized as described previously [20], Calibration standards were used to internally calibrate each sample over the m/z range 100 – 1000 amu as detailed in earlier work [20], FTICR data were analyzed using a linear least square regression line such that the internal standard peak had a mass error of < 1 ppm when compared to the theoretical mass. A peaklist containing the accurate mass and absolute intensity of each ion was created for the mass spectra from each analysis using XMASS software (Bruker Daltonics, MA, USA) as described previously [20]. A two dimensional array of mass versus ion intensity was created using DISCOVAmetrics software (Phenomenome Discoveries Inc, Saskatoon, Canada) and data were integrated from multiple files to determine unique masses. Processing and peak picking was carried out as described by Ritchie et al. [20] and produced a single data file per sample that was then merged and aligned to create a two dimensional metabolite array where rows represented metabolites and columns represented metabolite intensity values. The files generated contained the neutral mass of all of the 12C and higher intensity 13C metabolites. The intensities were expressed as a signal-to-noise (S/N) ratio. It also includes calibration statistics for validation purposes. Metabolite array tables were then used for statistical analyses.

### Statistical analysis

FTICR-MS accurate mass array alignments were performed using DISCOVAmetrics™ version 4.0, including principal component analysis (PCA), hierarchical clustering analysis (HCA) and unique PCA loading visualizations (Phenomenome Discoveries Inc, Saskatoon, Canada). Statistical analysis and graphs of FTICR-MS data were carried out using Microsoft Office Excel 2007. F-tests and Paired Student’s T-tests were used to assess significance with P-values of less than 0.05 considered significant. For every sample analyzed, there were a total of six analytical modes which were monitored for suppression effects and reproducibility through internal standards [20]. The coefficient of variation (CV) should average less than 20% per mode. In most cases the CV average was between 10 and 15%. Analytical reproducibility was monitored by analyzing a pooled CSF extract six times within each run. Each of the replicate profiles were then compared to each other by plotting the intensities of all combinations of the six replicates. R-squared values are typically > 0.95 (using non-log transformed data results in R-squared values > 0.999).

## Results

### Pathological characterization of the rabbit model used in this study

**Figure 1:**
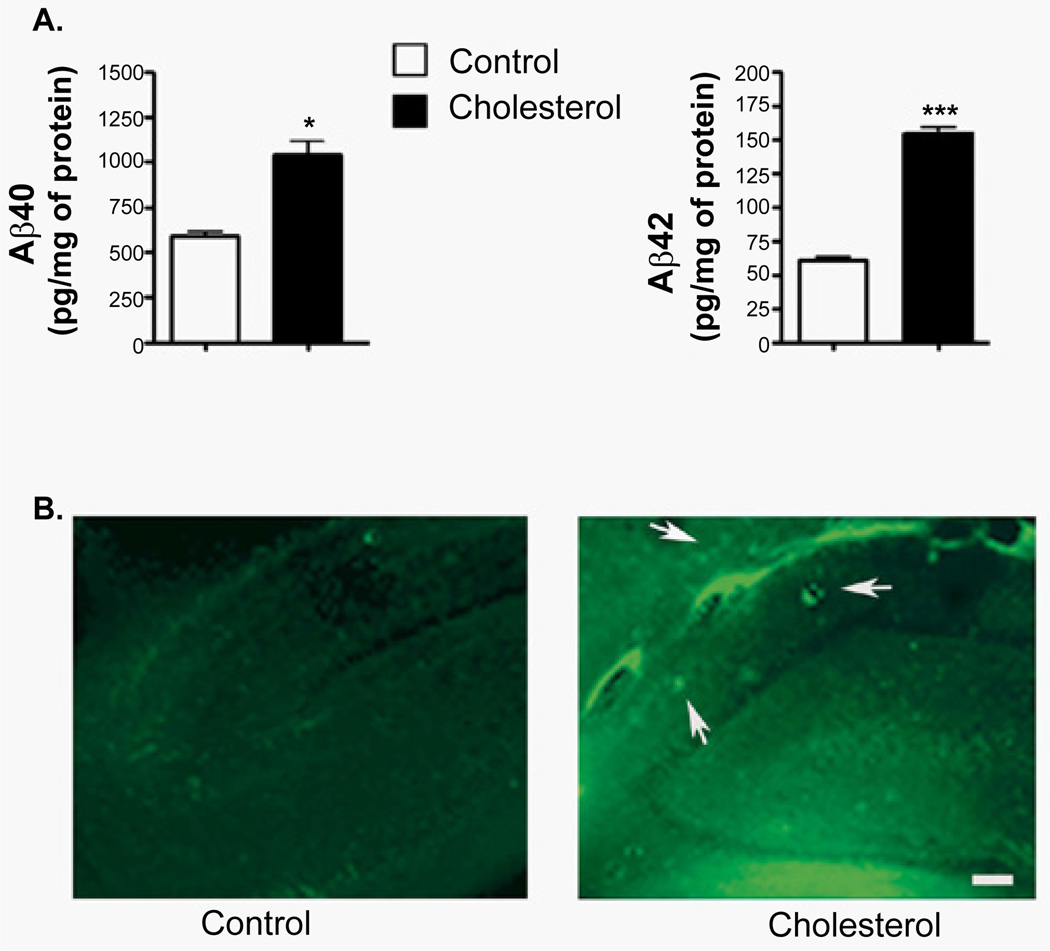
Cholesterol-enriched diet induced the accumulation of A β-like plaques in rabbit brain **A**. ELISA assay showed that the 2% cholesterol-enriched diet significantly increased both Aβ42 and Aβ40 levels. Values from control (n = 6) and 12 week cholesterol-fed (n = 6) rabbits were expressed as mean ± standard deviation, *p<0.05, ***p<0.01. **B**. While Thioflavin staining revealed no immunoreactivity to Aβ42 in control rabbits, Aβ42-like plaques was present in hippocampus of 12 week cholesterol-fed rabbits (arrows) as observed under fluorescence microscope. Scale bar = 50 µm.

To ensure that the rabbits fed with diet supplemented with 2% cholesterol develop AD characteristics, we performed ELISA analysis of the cortex from control and 12 week cholesterol treated rabbits (Figure 1A). This analysis confirmed that the treated rabbits had significantly higher amyloid load (Aβ 40 and Aβ 42) in their brains. Next, we performed immunohistochemical staining for Aβ plaques in the hippocampus from controls and cholesterol treated rabbits using Thioflavin S staining. Only sections from 12 week cholesterol treated rabbits showed Aβ like-plaques (Figure 1B, arrows).

### Global statistical overview of the metabolomic dataset

**Figure 2:**
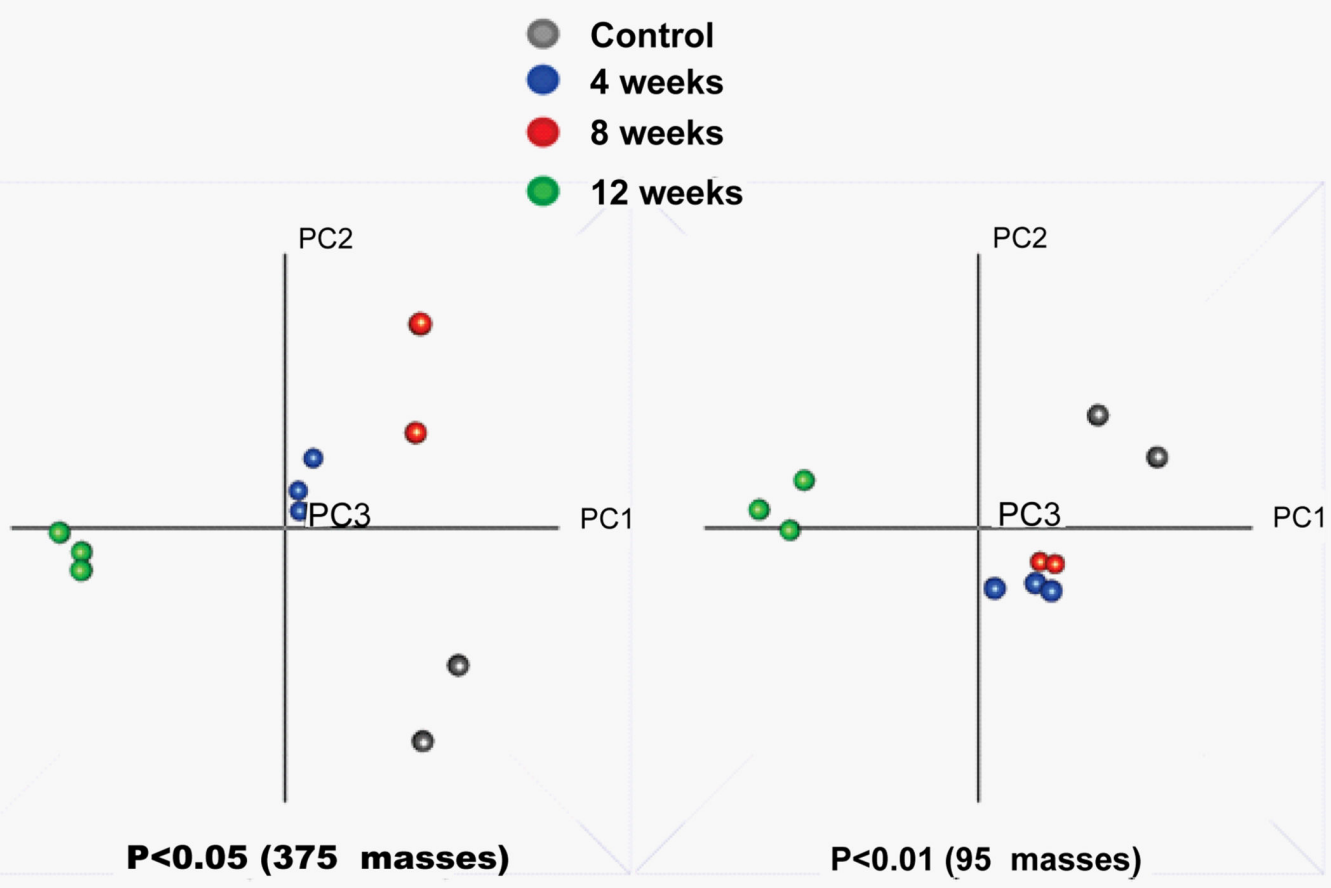
Principal component analysis plots (PCA) of all statistically significant masses This analysis was based on the data from an F-test comparison of all samples with a p-value < 0.05 (left panel, 375 masses, log_2_ scaled) and a p-value < 0.01 (right panel, 95 masses, log_2_ scaled). Groups are indicated in the legend between the PCA plots. Separation was observed between early (control to 8 weeks) and late time points (12 weeks) along PC1. Controls and week 4, 8 samples were separated along PC2.

Non-targeted metabolomic profiles of CSF from rabbits fed a high cholesterol diet over a twelve week time-course and control animals were generated. CSF metabolites (100–1500 Da) were captured through liquid extraction and infused directly into an FTICR mass spectrometer, using either electrospray ionization or atmospheric pressure ionization. Alignment of the mass spectrometry profiles of each sample allowed generation of two-dimensional metabolite arrays that were used for global statistical analyses. In total, the non-targeted metabolomic analysis of these CSF samples resulted in the detection of 6515 accurate masses. Table 1 summarizes the metabolomic dataset in terms of the total number of mass detected across each of the six analysis modes. A total of 3987 masses were detected from the two aqueous extractions and 2528 masses from four organic metabolite extractions. A principal components analysis (PCA) based on 375 masses with an F-test p-value less than 0.05, revealed a clear separation of the samples collected at the 4 time points (Figure 2 left panel). The PCA created using a more stringent cut-off with a p-value less than 0.01, 95 masses, revealed three clusters of samples (Figure 2 right panel). Samples collected at 12 weeks were clearly separated from the other time points along PCI, indicating that the greatest metabolic variance occurred between the early collections (controls, weeks 4 and 8) and the later collections (weeks 12). Although samples from the same time points are well clustered together, the week 4 and week 8 samples appeared metabolically very similar.

### Identification of masses correlating with cholesterol treatment

To begin identifying masses specifically associated with the diet, pair-wise t-tests were completed between each of the time points (Table 2). The number of significant masses increased with the length of time the rabbits were fed the cholesterol diet, with 382 masses with p < 0.05 present at week 4 versus control and 595 masses (p < 0.05) between 8 and 12 week samples.

**Figure 3:**
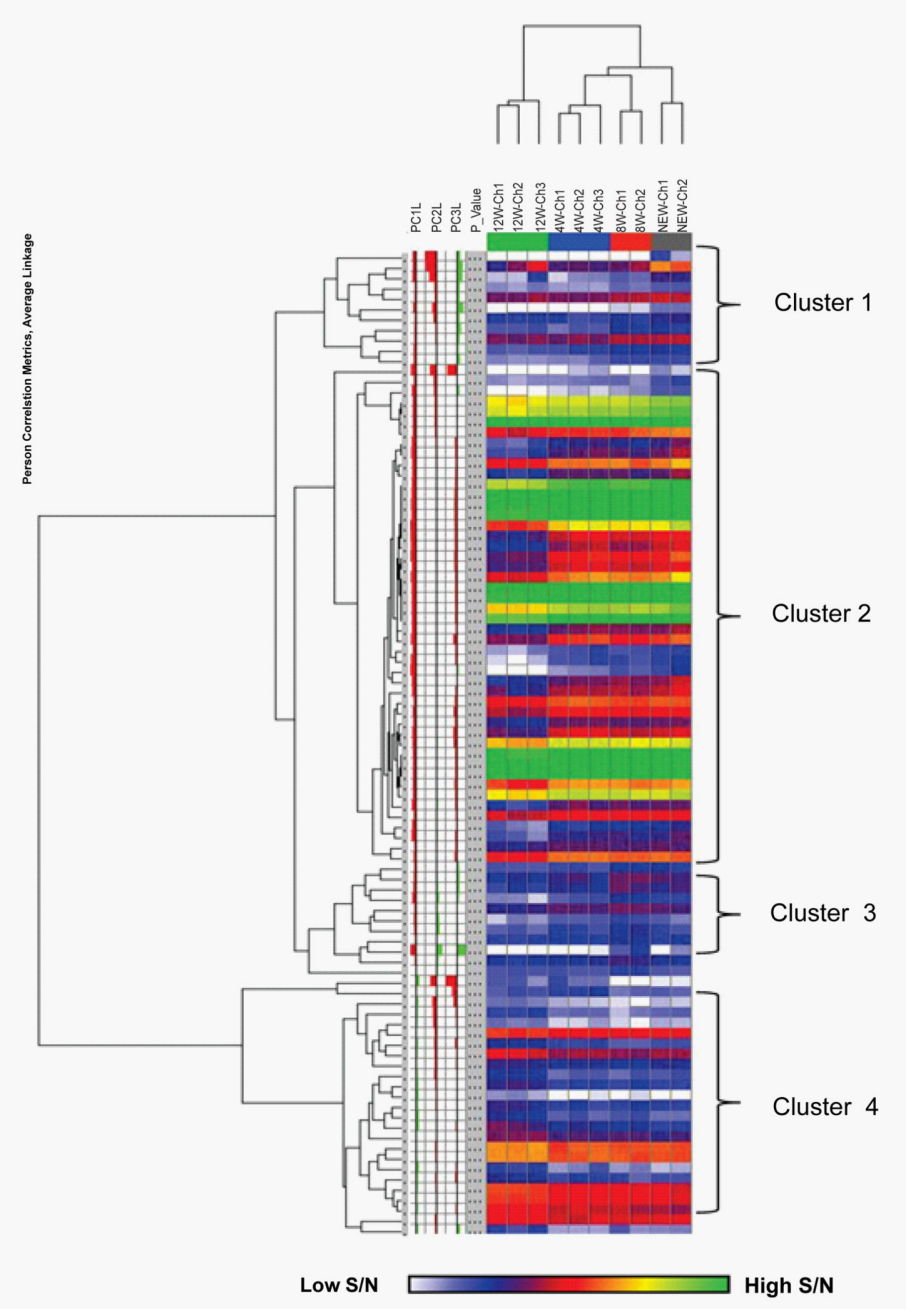
A hierarchically clustered two-dimensional heatmap (HCA) of the metabolite Intensities This HCA analysis was based on 95 masses showing significant differences with p value < 0.01. Cells are colored according to the signal to noise (SIN) intensity (log_2_ scaled). Masses clustered using a Pearson correlation, samples clustered using Euclidean correlation. Light blue - low intensity, dark blue/purple/red - medium intensity, orange/yellow/green - highest intensity.

A hierarchically clustered two-dimensional heatmap (HCA) of the metabolite intensities (p < 0.01, log2 scaled) is shown in Figure 3. Relative loadings contributions of each mass for principal components one through three are shown as red and green bar graphs on the left hand side of the HCA plot. Samples were clustered using a Euclidean distance metric and masses were clustered using a Pearson correlation coefficient distance metric, which produces clusters of masses based on similarity of expression from sample to sample. Similar to the PCA, complete separation was achieved between the four different collection time points, with the 12 week samples clustering separately from the other samples. The masses clustered into four distinct groups, as identified on the right side of the figure. The masses in the first 3 clusters all appeared to be detected at lower intensities in the samples collected at 12 weeks, and the fourth cluster contains masses that were elevated at 12 weeks.

**Figure 4:**
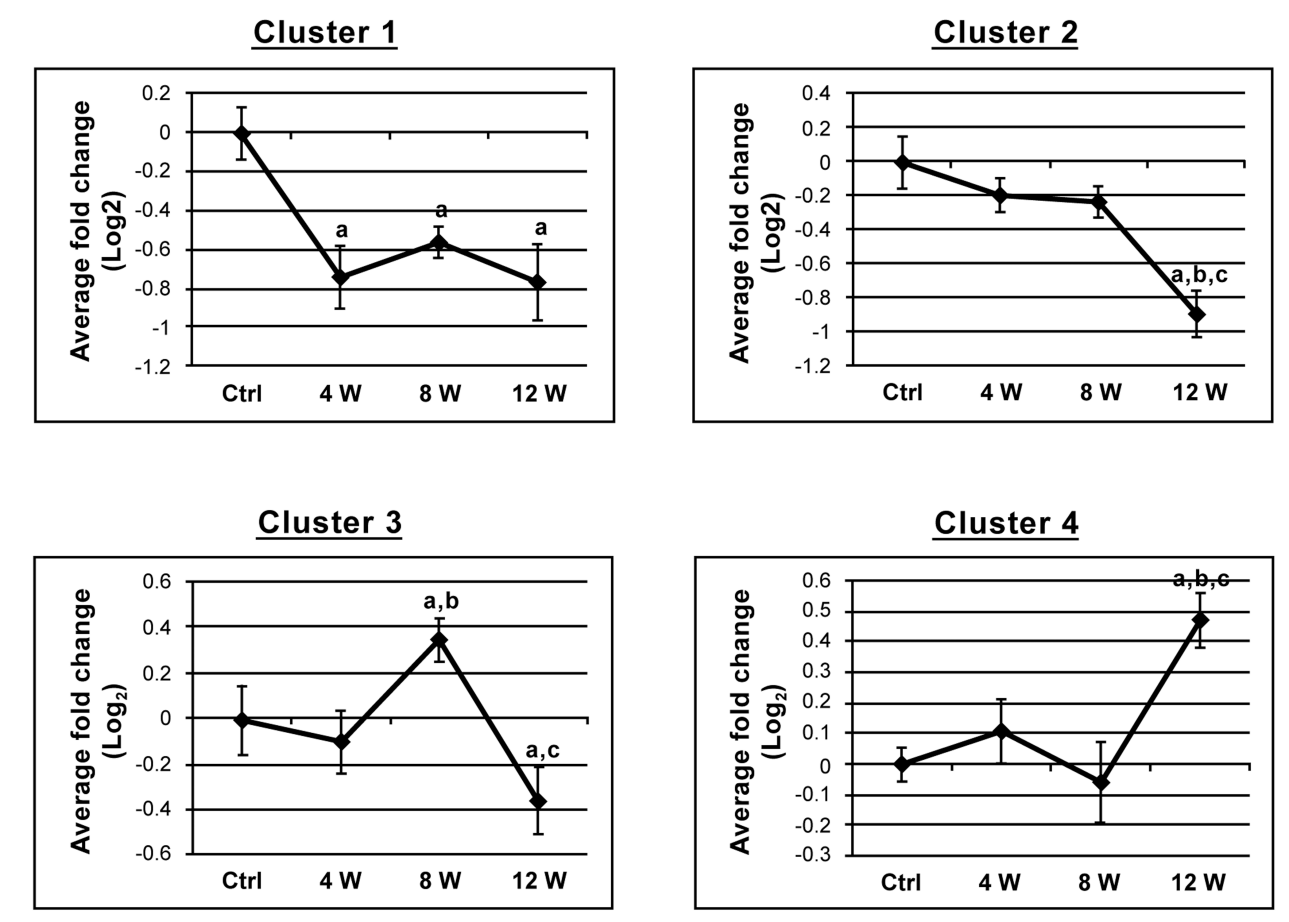
Graphs showing patterns of metabolic changes during the course of cholesterol treatment Graphs represent the average fold change (log_2_ scale) from controls for all masses contained within each of the four clusters identified in the HCA. Error bars represent standard deviations, a = p < 0.05 from control, b = p < 0.05 from week 4, c = p < 0.05 from week 8.

In order to identify the specific trend in each cluster, the data was normalized to the control samples and the average fold change across all of the masses was calculated and plotted (Figure 4). The masses in the first cluster were significantly (p < 0.05) lower in the samples collected from animals fed the high cholesterol diet (weeks 4, 8 and 12) compared to levels detected in the control animals. The masses in the second cluster were detected at lower levels in the week 12 samples in comparison to all other samples. The masses in the third cluster were detected at elevated levels in the 8 week samples (in comparison to the control and week 4 samples), and decreased in the week 12 samples (in comparison to the control and week 8 samples). The fourth cluster contained all masses that have been detected at significantly elevated levels in the week 12 samples in comparison to all other samples.

### Putative Metabolite Identifications

Masses within each cluster were further investigated by attempting to assign putative identifications using computational molecular formulas assignments and database searching in combination with contextual data such as ionization mode, extraction method, statistical clustering and biological relevance.

A number of phosphorylated glycerolipids have been identified in the second cluster of the HCA (Table 3). These masses are significantly decreased in the samples collected after 8 weeks of treatment. Two masses, 706.5527 and 674.5236, have been putatively identified as plasmanic acids, which are precursors for ether lipids such as plasmalogens. These lipids are likely all precursors, or breakdown products, of larger membrane phospholipids. The second and third clusters of the HCA contain a number of masses that have been putatively identified as peptide fragments, including post-translationally modified and multiply charged peptide fragments. The putative molecular formula assignments and metabolite identifications of these peptide fragments are summarized in Table 4. They are likely peptide fragments ranging in length from 4–8 amino acids. It is important to note that the identifications in the table represent putative amino acid assignments; however, it is not possible using this analytical method to determine their order.

Many of the masses detected in this study, cluster 1 and 4 in particular, do not correspond to known metabolites in publicly accessible databases, thus representing novel masses that have been uniquely detected in the CSF of this rabbit model. Since these masses have not been detected before, further analysis by MS-MS or NMR will be required in order to correctly determine their formula and identity.

## Discussion

Cholesterol fed rabbits has been adopted as a model for sporadic late-onset Alzheimer’s disease. Although pathological aspects of this model have been characterized [14–18], systematic studies on the biochemical changes including transcriptomics, proteomics and metabolomics have yet to be reported. We report here a study of the effect of dietary cholesterol on the metabolomic profile of rabbit CSF over a course of 12 weeks using high resolution FTICR-MS coupled with flow injection technology. Our data indicates that dietary cholesterol has a profound impact on the composition of metabolites in the CSF. Metabolomic differences were detected as early as four weeks after the high cholesterol diet was initiated with more dramatic metabolic changes occurring after 8 weeks of cholesterol treatment. The number of effected masses continued to increase with the length of time the rabbits were fed the enriched cholesterol diet. These findings are in accordance with the pathological properties of this model; rabbits have developed AD-like pathology by 10 weeks characterized in part by the detection of Aβ peptide (10 weeks) and hyperphosphorylated tau protein (12 weeks) in the brain [14,18]. There are a large number of masses detected in this model that have not been reported to be detected in blood and do not match any known metabolites in the databases that were interrogated [20]. This suggests that these novel masses may be unique to CSF and brain tissue and represent interesting targets for analyzing AD pathology. Identification of these currently unknown metabolites from accurate mass values alone is hampered by the lack of knowledge of CSF metabolites for humans or other animals. Only a limited number of studies have reported metabolomic profiling of ‘normal’ metabolite concentration ranges in human CSF [21].

Cholesterol, as either free cholesterol or cholesterol esters, is present in two pools in the body, which are separated by the blood brain barrier. Cholesterol is synthesized *de novo* in the brain, is present as free cholesterol in myelin, and is an integral component of plasma membranes [22–24]. By contrast, free cholesterol in the circulation is transported by lipoproteins (for example LDL, HDL, VLDL). Cholesterol does not have the ability to cross the blood brain barrier in healthy animals due to the impermeability of the blood brain barrier to proteins that carry cholesterol in the circulation. Therefore a direct link between dietary cholesterol, CSF cholesterol metabolites, and LOAD is not immediately apparent. This is especially true because brain cholesterol levels remain unchanged in rabbits that are fed a cholesterol rich diet [18]. However, an over accumulation of cholesterol in hippocampal neurons has been observed due to focal disruption of the BBB and redistribution of cholesterol within brain cells [16,17]. Cholesterol breakdown metabolites, such as 27-hydroxycholesterol, and (24S)-hydroxycholesterol do have the ability to cross the blood brain barrier. We have shown that it is 27-hydroxycholesterol that enhances the production of Aβ by up-regulating APP42 in SH-SY5Y cells [25]. It was therefore proposed that 27-hydroxycholesterol is likely the link between circulating cholesterol and AD like brain pathology [18]. Interestingly, none of the changed masses identified as significant in the rabbit CSF matches the cholesterol metabolites such as 27-hydroxycholesterol, or (24S)-hydroxycholesterol, suggesting that these oxysterols might have been further metabolized or converted into others molecules secreted into the CSF.

In the present study, the significantly affected masses could be separated into four clusters based on how they varied throughout the time-course of the experiment. The first cluster represented a small number of metabolites with decreased levels observed at 4 weeks on the cholesterol diet which remained significantly decreased for the remainder of the 12 week treatment. These masses are likely directly associated with high cholesterol content in the diet. Masses which are present in the second cluster remain unchanged throughout the first 8 weeks of treatment but undergo a significant decrease at the 12 week time-point. Identification of these masses suggested that the majority belong to a common family including phosphorylated fatty alcohols, akylacyl or dialkyl-glycerophosphates (Table 3). All of these metabolites are potential precursors or degradation products of phospholipids including phosphatidylcholines and plasmalogens. Phospholipids and their precursors are largely synthesized in the liver and then excreted from cells into lipoproteins (predominantly HDL and LDL) for transport throughout the body where they are incorporated into the cellular membrane [26]. Transport of phospholipids into the CSF has been shown to occur by receptor-mediated transcytosis of LDL transporters [27,28]. Hepatic damage is also known to occur after long term cholesterol loading [29]. Phosphatidylcholine and phosphatidylethanolamine levels were shown to be decreased in the liver after cholesterol-loading suggesting that liver damage may affect its ability to produce phospholipids. A decrease in the concentrations of circulating LDL-phopholipid was also reported [30]. These previous results suggest that the observed decrease of fatty alcohols, alkylacyl and dialkyl-glycerophosphates in the CSF could result from either altered transport from the liver to the CSF or decreased liver synthesis, or a combination of both processes. These findings are in agreement with a previous report showing a correlation between decreased liver phospholipid metabolism and Alzheimer’s disease [31],

The observed decrease in these possible precursors of phospholipid synthesis at week 12 could represent a change in the membrane composition of cells within the brain. Increased membrane cholesterol causes a shift in the composition of the cellular membrane to include more cholesterol-rich lipid rafts. A clear link exists between membrane cholesterol levels, increased dietary cholesterol and the production of Aβ and AD pathogenesis [8–11,32,33]. Alpha-secretase is located in phospholipid-rich regions of the cell membrane and performs non-pathological APP processing, while β-secretase is located in lipid rafts and is involved in the processing of APP into Aβ. Increased membrane cholesterol has been shown to decrease α-secretase activity [34] and increase β-secretase activity which explains the increased production of Aβ [35].

Only after 12-weeks of cholesterol treatment did animals show Aβ like-plaques (Figure 1B, arrows). This is in agreement with the decrease in phospholipid precursors only at this time point, suggesting a shift in membrane composition towards increased lipid rafts, which would lead to increased β-secretase activity and Aβ productions resulting in the observed Aβ like-plaques. Further MS-MS analysis of these masses could confirm their identities and may allow them to be used as CSF markers of AD pathology. One successful example of such a metabolite is the discovery of the negative correlation of serum plasmalogen with age and its link with altered cholesterol processing and AD [36,37]. Plasmalogens are currently being explored as AD diagnostic markers and its synthetic precursor compounds are being tested as potential therapeutics for AD [37].

Putative identifications of masses revealed a number of peptide fragments decreased after 8 weeks of cholesterol treatment, some of them are transiently increased at the 8 week time-points and then undergo a significant decline at week 12 to levels lower than control. These results suggest that cholesterol treatment causes significant protein expression changes in the rabbit brain. The proteins from which these peptide fragments are derived are currently unknown. In order to determine which proteins are potentially being affected by the cholesterol treatment, analysis using MALDI-TOF MS-MS would need to be performed to determine the precise sequence of the amino acids, which could provide insight into the link between cholesterol treatment and AD pathology. Cluster 4 was the only group that showed a significant increase in levels at the 12 week time-point but was unaltered at any of the earlier points. Majority of masses in this group are novel metabolites. Follow up MS-MS study is required to determine their identities.

CSF may be a useful fluid for the study of neurodegenerative diseases because it is close to the site of pathology and more directly reflects the metabolic state of the brain due to the free exchange of molecules between the brain and the CSF. However, animals must be euthanized before collection and scarified after collection in order to collect sufficient volume of clean CSF samples. One technical difficulty encountered during this study was blood contamination into the CSF of two rabbits, resulting in n=2 for two variables. However, the percentage of CVs between the replicates on average was very good, which provided good confidence even in the time points with only two replicates.

## Conclusions

We have performed a non-targeted metabolomic analysis of the CSF from a rabbit LOAD model generated by feeding a cholesterol-enriched diet. The objectives were to further characterize this model at the molecular level and identify biomarkers for LOAD. This study revealed a number of metabolites that were either responsive to cholesterol or change as a consequence of brain degeneration caused by high cholesterol levels. We are in the process of further identifying these masses and the molecular pathways in which they participate. It is hoped that follow up investigations of these masses will help us understand the molecular mechanisms of cholesterol-related AD development. These metabolites, or their derivatives, may be potential LOAD diagnostic, prognostic or theranostic markers.

## Figures and Tables

**Table 1:** Extraction and ionization mode and detected masses per analytical mode.

Mode	Ionization Mode	Extract	Number of Detected Masses
**1101**	Positive	Aqueous	1059
**1102**	Negative	Aqueous	2928
**1201**	Positive	Organic 1	513
**1202**	Negative	Organic 1	1550
**1203**	Positive	Organic 2	224
**1204**	Negative	Organic 2	241
**Total**			6515

**Table 2:** The number of masses deemed statistically significant from t-test comparisons between each pair of samples.

p<0.05	Control	Week 4	Week 8	Week 12
**Control**		382	286	449
**Week 4**			193	339
**Week 8**				595
**Week 12**				

**Table 3:** Putative molecular formula assignments and metabolite identifications for a group of fatty alcohol phosphate decreased after 8 weeks of treatment.

					Average S/N (log2)			
Detected Mass	Analysis Mode	P_Value	Formula	Metabolite	Ctrl	4week	8week	12week
394.2846	1102	0.0015	C_20_H_43_O_5_P	Possible Phosphorylated Fatty alcohol	9.6	9.55	9.49	9.01
422.3158	1102	0.0003	C_22_H_47_O_5_P	Possible Phosphorylated Fatty alcohol	5.67	5.76	5.6	4.98
450.3471	1102	0.0024	C_24_H_51_O_5_P	Possible Phosphorylated Fatty alcohol	3.15	3.11	3.11	2.28
410.2793	1102	0.009	C_20_H_43_O_6_P	1-alkyl-2-lyso-glycerophosphate (1-O-17:0/OH)	3.79	3.56	3.52	2.91
632.5132	1102	0.0003	C_36_H_73_O_6_P	1,2-dialkyl-glycerophosphate (1-O-16:1/2-O-17:0)	9.2	9	9	8.2
646.5281	1102	0.0002	C_37_H_75_O_6_P	1,2-dialkyl-glycerophosphate (1-O-16:1/2-O-18:0)	4.4	4.2	4	3.1
660.5457	1102	0.0002	C_38_H_77_O_6_P	1,2-dialkyl-glycerophosphate (1-O-16:1/2-O-19:0)	9.7	9.5	9.4	8.5
706.5527	1102	0.0007	C_39_H_79_O_8_P	1-alkyl-2-acyl-glycerophophate (1-O-16:0/20:0) (OH)	3.4	3.3	3.3	2.5
618.498	1102	0.0048	C_35_H_71_O_6_P	1,2-dialkyl-glycerophosphate (1-O-16:1/2-O-16:1)	3.1	2.8	2.7	2
674.5236	1102	0.0021	C_38_H_75_O_7_P	1-alkyl-2-acyl-glycerophophate (1-O-16:0/19:0)	3.6	3.2	3.4	2.6

**Table 4:** Putative molecular formula assignments and metabolite identifications for peptide fragments changed due to cholesterol treatment.

						Average S/N (log2)			
Cluster	Detected Mass	Analysis Mode	P_Value	Formula	Metabolite ID	Ctrl	4week	8week	12week
2	1030.579	1102	0.0003	C_48_H_78_N_12_O_13_	L-lsoleucyl-L-leucyl-L-asparaginyl-L- histidyl-L-isoleucyl-L-valyl-L-prolyl-L- prolyl-L-glutamic acid	2.4	1.7	2.0	0.8
2	674.2703	1102	0.0022	C_34_H_38_N_6_O_9_	Glycyl-L-tyrosyl-L-tyrosyl-L-tryptophyl- L-serine	6.2	5.8	5.9	5.4
2	672.2733	1102	0.0039	C_31_H_40_N_6_O_11_	L-a-Aspartyl-L-tyrosyl-L-tyrosyl-L- valyl-L-asparagine	8.0	7.5	7.6	7.2
2	704.3001	1102	0.0093	C_28_H_48_N_8_O_9_S_2_	L-Cysteinyl-L-alanyl-L-valyl-L-valyl-L- prolyl-L-asparaginyl-L-cysteine	4.2	4.1	4.1	3.7
2	398.288	1102	0.004	C_20_H_38_N_4_O_4_	N-Acetyl-L-leucyl-L-leucyl-L- norleucinamid	2.7	2.4	2.5	1.8
3	918.3372	1102	0.0025	C_43_H_50_N_8_O_15_	Glycyl-L-a-aspartyl-L-a-glutamyl-L- tyrosyl-L-tyrosyl-L-tryptophyl-L-serine	3.0	3.1	3.2	2.8
3	817.4655	1101	0.005	C_34_H_63_N_11_O_12_	Glycy l-L-seryl-L-seryl-L-lysyl-L-seryl-L- lysyl-L-prolyl-L-lysine	2.4	2.4	2.6	2.3
